# IMU Signal Generator Based on Dual Quaternion Interpolation for Integration Simulation

**DOI:** 10.3390/s18082721

**Published:** 2018-08-18

**Authors:** Ke Liu, Wenqi Wu, Kanghua Tang, Lei He

**Affiliations:** 1 College of Artificial Intelligence, National University of Defense Technology, Changsha 410073, China; wenqiwu_lit@hotmail.com(W.W.); tt_kanghua@hotmail.com (K.T.); 2 College of Systems Engineering, National University of Defense Technology, Changsha 410073, China; helei_nudt@163.com

**Keywords:** IMU signal generator, dual quaternion, integration simulation, spline interpolation

## Abstract

This paper focuses on the problem of high-update-rate and high accuracy inertial measurement unit signal generation. In order to be in accordance with the vehicle’s kinematic and dynamic characteristics as well as the characteristics of pseudorange of post-processed global navigation satellite system and their rate measurements, a novel dual quaternion interpolation and analytic integration algorithm based on actual flight data is proposed. The proposed method can simplify the piecewise analytical expressions of angular rates, angular increments and specific force integral increments. Norm corrections are adopted as constraint conditions to guarantee the accuracy of the signals. Numerical simulations are conducted to validate the method’s performance.

## 1. Introduction

Inertial measurement unit (IMU) signal generators have been used in numerous engineering fields for algorithm testing and verification to reduce experimental costs and shorten developing time under laboratory conditions. It can be used in industrial robots for motion control [1,2,3], path planning of unmanned aerial vehicles (UAV) [4], tightly coupled integrated navigation research [5,6,7] and guidance system simulation [8]. A Global Navigation Satellite System (GNSS) signal simulator is required to generate multiple synchronous signals, one for each satellite in view, and to generate consistent navigation data for the receiver to be able to compute the position. Moreover, a corresponding IMU signal simulator is also necessary to provide gyro and accelerometer measurements for the strapdown inertial navigation system (SINS) and GNSS integration research.

The followings are required for a IMU signal generator to be used in the tightly coupled SINS/GNSS integration simulation:

(1) GNSS’s pseudorange and their rate measurements corresponding to the simulated navigation parameters such as position and velocity as well as satellite ephemeris are required.
(2) The simulated gyro measurements and accelerometer measurements in the trajectory are coincident with the vehicle’s kinematic and dynamic characteristics.
(3) The IMU size effects as well as the level arm effects caused by the displacements of the GNSS receiver antenna from the IMU reference point can be simulated. For this purpose, the analytic angular rate and the angular acceleration data are required.
(4) Various SINS algorithms and update rates can be evaluated under the dynamic environment in trajectories through simulations.

In this paper, an actual-flight-data based IMU signal generation algorithm is presented. Actual GNSS navigation data files (including satellite ephemeris) and observation data files (including GNSS’s pseudorange and their rate measurements) can be obtained through practical flight experiments. The post-processed GNSS navigation solution and a low-cost loosely coupled SINS/GNSS integrated navigation system provide position, velocity and attitude parameters at discrete time intervals (1 Hz). The spline interpolation method is used to generate analytic continuous-time kinematic simulation trajectory. The main contributions of this paper can be summarized as follows: (1) A novel IMU signal generation algorithm is proposed based on dual quaternions and actual flight data. (2) The effectiveness and efficiency of the proposed algorithm are validated by extensive simulation experiments. (3) Compared with state-of-the-art methods, the proposed method generates signals with the highest accuracy in a relatively short computation time.

This paper is organized as follows. Section 2 introduces the related works about the IMU signal generator and dual quaternions. Section 3 provides a dual quaternion representation of angular increments and specific force integral increments for IMU measurements simulation. Section 4 provides two-point piecewise cubic spline interpolation, analytic integration, norm corrections and error analysis of interpolated dual quaternions. Simulation tests for analytic IMU signal generator validation are presented in Section 5. Conclusions are drawn in Section 6.

## 2. Related Works

For the IMU signal generator, over the decades, various approaches have been developed, which can be classified into three general categories: pure mathematical models [9,10,11,12,13], kinematic or dynamic models [14,15,16,17] and actual flight-data based methods [18,19].

The methods based on pure mathematical models mainly depend on the vehicle’s straightforward manoeuvre, such as climbing up, straight flight, turning flight and diving. Moreover, the integration of these manoeuvres can be simulated. Two types of highly straightforward models are commonly used. The first one is the linear acceleration that has phase step with 50 g, wherein the acceleration is a constant. The other one is the circular motion or turn that has radial acceleration with 50 g; furthermore, all the velocities and derivations follow the sinusoidal waveform. Although the form is adopted, all of them are highly straightforward. A trajectory generated by these methods can neither reflect the complex kinematics and dynamics of a vehicle nor be conducive to combining with actual flight experiments to produce consistent simulation trajectory using satellite signals and multi-sensor measurement data such as those from image sensors. In kinematic and dynamic model based methods, classical kinematic or dynamic equations are used to generate inertial sensor measurements under flight simulation conditions, and the impact of the pneumatic environment of a vehicle can also be considered. The drawback is that it is generally challenging to predetermine the position and attitude of the vehicle precisely in the simulated trajectory. In addition, physical and mathematical models are often excessively complex to be described, and the degree of approximation between the generated trajectory and the vehicle’s actual motion depends on the accuracy of the kinematic models. Based on actual flight data, the incremental outputs of the inertial sensors are inversely deduced from the attitude, velocity and positioning information using a numerical strapdown inertial navigation system (SINS) attitude and velocity updating algorithm, which can be referred to as an inverse SINS algorithm. Generally, this algorithm requires high update rate of data of navigational parameters (100 Hz–1000 Hz). Because the inverse SINS algorithm is used, the accuracy influences of the strapdown inertial navigation algorithm and update rates are not considered. It is not feasible to simulate error factors such as size effects and lever arm effects because of the lack of the angular rate and the angular acceleration data in numerical simulations using the inversed SINS algorithm.

Dual quaternions (DQ) can provide a uniform representation of the rotational and translational motion of a rigid body in space. It was proposed by Clifford in 1873 and based on the dual quaternion algebra to describe the spiral variation. In 1992, dual quaternion was first used in the inertial navigation field by Branets and Shmyglevsky [20]. They analysed its feasibility and drew the outline of various coordinates’ relationships in SINS. In 2004, Y.X Wu elaborated upon strapdown inertial navigation system algorithms based on the dual quaternion and demonstrated the consistency of the differential equations between dual quaternion algorithms and traditional algorithms [21]. Compared with the general methods, the superior accuracy performance of the dual quaternion has been proved [21,22]. In [21], the author analysed the algorithm errors and proved that the algorithm based on DQ was superior to conventional ones in terms of the accuracy of the translational components (velocity vectors and position vectors) from both the analytic forms and the numerical simulation perspectives. Moreover, it was indicated that the new algorithm was a better choice than all conventional algorithms for applications in high-accuracy navigation systems and high-manoeuvre applications. In [22], the accuracy advantage of the algorithm based on DQ was analysed, and the analytic comparisons indicated that the thrust velocity solution as well as the attitude and gravitational velocity solution were superior to the traditional navigation algorithms in terms of accuracy.

In this study, a novel dual quaternion interpolation and analytic integration algorithm is used to generate high update rate and high accuracy IMU signals for actual-flight-data based integrated navigation simulation, in which the piecewise analytical expressions of angular increments and specific force integral increments in the body frame are simplified. A method to solve the norm constraint problems in quaternion and dual quaternion interpolation is presented.

## 3. Dual Quaternion Representation of Angular Increments and Specific Force Integral Increments

In this section, the relationship between dual quaternion and angular rates in the body frame and that between dual quaternion and specific force in the body frame are presented for ease of reference and for providing the foundation for the dual quaternion interpolation.

### 3.1. Rotation and Translation Expressed as a Dual Quaternion

Rotation and translation between the inertial frame (*i* frame) and the body frame (*b* frame) can be expressed as a dual quaternion in the following manner [21]

(1) $$\overset{⌢}{q}=q+\varepsilon p=q+\varepsilon \frac{1}{2}t^{i}\circ q=q+\varepsilon \frac{1}{2}q\circ t^{b}$$

where, $\overset{⌢}{q}=q+\varepsilon p$ is the dual quaternion, and ε^2^ = 0. The real part **q** is the general quaternion to facilitate the representation of rotation, **p** is the dual part of $\overset{⌢}{q}$, and ∘ represents the quaternion or dual quaternion multiplication. The translation component **t** can be defined as (see Appendix A for details)

(2) $$\left\{\begin{array}{c} t^{b}=q^{*}\circ \left(v_{ib}^{i}-\int_{0}^{t}g_{ib}^{i}dt\right)\circ q\\ t^{i}=v_{ib}^{i}-\int_{0}^{t}g_{ib}^{i}dt \end{array}\right.$$

where, $v_{ib}^{c}$ is the velocity vector of frame *b* relative to frame *i* expressed in frame *c*, $g_{ib}^{c}$ is the gravitational acceleration vector expressed in frame *c*.

The rotational and translational kinematics can be expressed as a differential equation of the dual quaternion as

(3) $$\dot{\overset{⌢}{q}}=\frac{1}{2}\overset{⌢}{q}\circ {\overset{⌢}{\omega }}_{ib}^{b}$$

in which

(4) $${\overset{⌢}{\omega }}_{ib}^{b}=\omega_{ib}^{b}+\varepsilon ({\overset{˙}{t}}^{b}+\omega_{ib}^{b}+t^{b})$$

where, ${\overset{⌢}{\omega }}_{ib}^{b}$ is a dual vector and generally referred to as twist, × represents the vector product, and ***ω***_*c*_1_*c*_2__ represents the angular rate vector of frame *c*_2_ relative to frame *c*_1_.

Using $2\dot{q}=\omega_{ib}^{i}\circ q=q\circ \omega_{ib}^{b}$ and the fact that $q\circ q^{*}=1$, where * represents the conjugate operator, the following is obtained:

(5) $$\begin{array}{c} {\dot{t}}^{b}+\omega_{ib}^{b}\times t^{b}=q^{*}\circ \left({\dot{v}}_{ib}^{i}-g_{ib}^{i}\right)\circ q+{\dot{q}}^{*}\circ \left(v_{ib}^{i}-\int_{0}^{t}g_{ib}^{i}dt\right)\circ q+q^{*}\circ \left(v_{ib}^{i}-\int_{0}^{t}g_{ib}^{i}dt\right)\circ \dot{q}+\omega_{ib}^{b}\times t^{b}\\ =q^{*}\circ \left(a_{ib}^{i}-g_{ib}^{i}\right)\circ q-q^{*}\circ \dot{q}\circ q^{*}\circ \left(v_{ib}^{i}-\int_{0}^{t}g_{ib}^{i}dt\right)\circ q+q^{*}\circ \left(v_{ib}^{i}-\int_{0}^{t}g_{ib}^{i}dt\right)\circ \dot{q}+\omega_{ib}^{b}\times t^{b}\\ =q^{*}\circ \left(a_{ib}^{i}-g_{ib}^{i}\right)\circ q+q^{*}\circ \left(t^{i}\times \omega_{ib}^{i}\right)\circ q+\omega_{ib}^{b}\times t^{b}\\ =q^{*}\circ f_{ib}^{i}\circ q=f_{ib}^{b} \end{array}$$

where $f_{ib}^{c}$ is the specific force vector expressed in frame *c*, $a_{ib}^{c}$ is the acceleration vector of frame *b* relative to frame *i* expressed in frame *c*.

Substituting Equation (5) in Equation (4), we obtain

(6) $${\overset{⌢}{\omega }}_{ib}^{b}=\omega_{ib}^{b}+\varepsilon f_{ib}^{b}$$

where the twist is a combination of the angular velocity and the specific force in frame *b*.

Substituting Equation (6) in Equation (3), the twist is obtained as follows:

(7) $$\begin{array}{cc} {\overset{⌢}{\omega }}_{ib}^{b} & =2{\overset{⌢}{q}}^{*}\circ \dot{\overset{⌢}{q}}=\omega_{ib}^{b}+\varepsilon f_{ib}^{b}\\ & =2{\left(q+\varepsilon p\right)}^{*}\circ (\overset{˙}{q}+\varepsilon \dot{p})\\ & =2q^{*}\circ \overset{˙}{q}+\varepsilon 2(q^{*}\circ \overset{˙}{p}+p^{*}\circ \overset{˙}{q}) \end{array}$$

### 3.2. Integral Increments of Angular Rates and Specific Force in Body Frame

Integrating Equation (7) over the sampling time interval $\left[t_{k}-\Delta T,t_{k}\right]$, the angular rate increments $\Delta \theta \left(t_{k}\right)$ and the specific force integral increments $\Delta \upsilon \left(t_{k}\right)$ at time $t_{k}$ can be expressed as

(8) $$\Delta \theta \left(t_{k}\right)=\int_{t_{k}-\Delta T}^{t_{k}}\omega_{ib}^{b}\left(t\right)dt=\int_{t_{k}-\Delta T}^{t_{k}}2\left[q^{*}\left(t\right)\circ \dot{q}\left(t\right)\right]dt=-2\int_{t_{k}-\Delta T}^{t_{k}}\left[{\dot{q}}^{*}\left(t\right)\circ q\left(t\right)\right]dt$$

(9) $$\begin{array}{cc} \Delta \upsilon \left(t_{k}\right) & =\int_{t_{k}-\Delta T}^{t_{k}}f_{ib}^{b}\left(t\right)dt=2\int_{t_{k}-\Delta T}^{t_{k}}\left[q^{*}\left(t\right)\circ \dot{p}\left(t\right)+p^{*}\left(t\right)\circ \dot{q}\left(t\right)\right]dt\\ & =2{\left.q^{*}\left(t\right)\circ p\left(t\right)\right|}_{t_{k}-\Delta T}^{t_{k}}-2\int_{t_{k}-\Delta T}^{t_{k}}\left[{\dot{q}}^{*}\left(t\right)\circ p\left(t\right)\right]dt+2\int_{t_{k}-\Delta T}^{t_{k}}\left[p^{*}\left(t\right)\circ \dot{q}\left(t\right)\right]dt\\ & =2{\left.q^{*}\left(t\right)\circ p\left(t\right)\right|}_{t_{k}-\Delta T}^{t_{k}}-4\int_{t_{k}-\Delta T}^{t_{k}}\left[{\dot{q}}^{*}\left(t\right)\circ p\left(t\right)\right]dt \end{array}$$

where

(10) $$\left\{\begin{array}{c} q^{*}\left(t\right)\circ \dot{q}\left(t\right)=-{\left[q^{*}\left(t\right)\circ \dot{q}\left(t\right)\right]}^{*}=-{\dot{q}}^{*}\left(t\right)\circ q\left(t\right)\\ p^{*}\left(t\right)\circ \dot{q}\left(t\right)=-{\left[p^{*}\left(t\right)\circ \dot{q}\left(t\right)\right]}^{*}=-{\dot{q}}^{*}\left(t\right)\circ p\left(t\right) \end{array}\right.$$

considering $q^{*}\left(t\right)\circ \dot{q}\left(t\right)$ and $p^{*}\left(t\right)\circ \dot{q}\left(t\right)$ are quaternions with zero scalar parts.

## 4. Piecewise Constraint Interpolation and Analytic Integration Algorithm

In this section, a novel IMU signal generation algorithm is developed based on the dual quaternion interpolation, which is suitable for implementation in navigation fields. The two-point piecewise cubic spline interpolation and the analytic integration are used for the dual quaternion, and angular increments and specific force increments can be obtained. Additionally, the error analysis and methods for accuracy enhancement are also presented.

**Figure 1:**
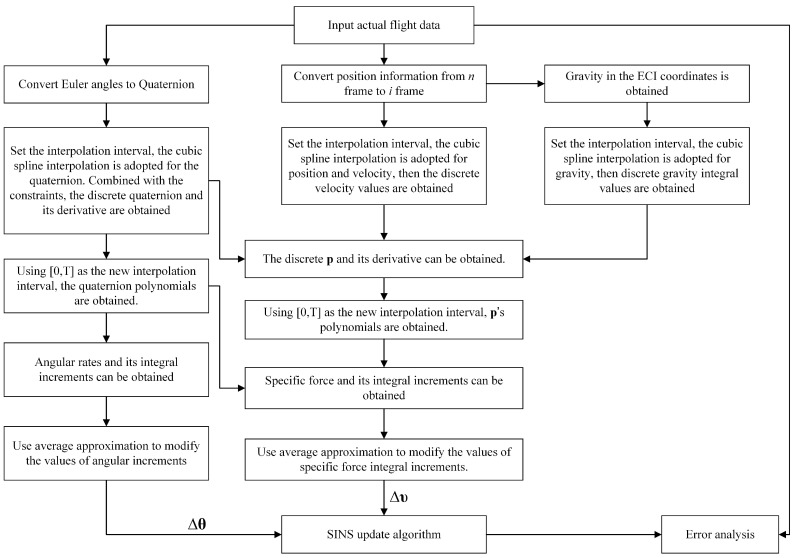
Algorithm flow chart.

An algorithm flow chart is illustrated in Figure 1 that provides the detailed steps to streamline the algorithm proposed in this paper. The proposed method provides a necessary basis for the offline test of the performance of SINS and its fusion with other sensors’ signals to verify the performance of the integrated navigation algorithm. By using this algorithm, we can get IMU signal data with any frequency. If the sensor (IMU) outputs and all the flight data can be collected, the proposed algorithm is not necessary.

The algorithm can be divided into the following three parts:

- Attitude Part
  (1) Convert Euler angles (roll, pitch and yaw) from the navigation frame *n* to quaternion in frame *i*.
  (2) Set the interpolation interval $T_{interp}$. The cubic spline interpolation is adopted for the quaternion. Combined with the constraint conditions, the discrete quaternion q and its derivative are obtained.
  (3) Using $\left[0,T\right]$ as the new interpolation interval, the polynomial of $q\left(t\right)$ are obtained.
  (4) Calculate the angular rate and its integral increments.
  (5) Use average approximation to modify the values of angular increments.
- Position Part
  (1) Convert the position information (latitude, longitude and altitude) from the navigation frame *n* to the inertial frame *i*.
  (2) Set the interpolation interval $T_{interp}$. The cubic spline interpolation is adopted for the position and velocity. Then, the discrete velocity values can be obtained.
  (3) Gravity values in the Earth-centered inertial (ECI) coordinates are calculated according to the gravity formula.
  (4) Set the interpolation interval $T_{interp}$. The cubic spline interpolation is adopted for the gravity values. Then, discrete gravity integral values can also be obtained.
  (5) Using the discrete quaternion and its derivative, discrete velocity values and its derivative, discrete gravity and its integral values, discrete p and its derivative can be obtained.
  (6) Using $\left[0,T\right]$ as the new interpolation interval, the polynomials of $p\left(t\right)$ are obtained.
  (7) Combined with the polynomials of $q\left(t\right)$, the specific force and its integral increments are obtained.
  (8) Use average approximation to modify the values of specific force integral increments.
- Solution Part

Based on the two sub-sample error compensation algorithms, the inertial sensor simulated samples Δυ and Δθ are used to update pure SINS. The attitude and the position results are compared with the actual flight data input.

In this algorithm, two sample time intervals are involved. The first one is the interpolation time interval, which is the interval between the interpolation points selected from the original input data. The second one is the integration time interval, which is the interval for the integration of angular rate and specific force.

### 4.1. Dual Quaternion Calculation at Discrete Time Based on Actual Flight Data

The actual flight data including navigational parameters such as position $r_{ib}^{i}$, velocity $v_{ib}^{i}$ and quaternion q at discrete time $t_{m}$ can be obtained through a SINS/GNSS loosely integrated navigation system in a UAV.

The dual quaternion and its derivative at discrete time $t_{m}$ can be calculated based on the following equations:

(11) $$\begin{array}{cc} \overset{⌢}{q}\left(t_{m}\right) & =q\left(t_{m}\right)+\varepsilon p\left(t_{m}\right)\\ & =q\left(t_{m}\right)+\varepsilon \frac{1}{2}\left(v_{ib}^{i}\left(t_{m}\right)-\int_{t_{0}}^{t_{m}}g_{ib}^{i}dt\right)\circ q\left(t_{m}\right) \end{array}$$

(12) $$\begin{array}{cc} \dot{\overset{⌢}{\;q}}\left(t_{m}\right) & =\dot{q}\left(t_{m}\right)+\varepsilon \dot{p}\left(t_{m}\right)\\ & =\dot{q}\left(t_{m}\right)+\varepsilon \frac{1}{2}\left(v_{ib}^{i}\left(t_{m}\right)-\int_{t_{0}}^{t_{m}}g_{ib}^{i}dt\right)\circ \dot{q}\left(t_{m}\right)+\varepsilon \frac{1}{2}\left({\dot{v}}_{ib}^{i}\left(t_{m}\right)-g_{ib}^{i}\right)\circ q\left(t_{m}\right) \end{array}$$

where, $g_{ib}^{i}$ is the function of $r_{ib}^{i}$. The gravitation integration is calculated as follows:

(13) $$\int_{t_{0}}^{t_{m}}g_{ib}^{i}dt=\int_{t_{0}}^{t_{m}}g_{ib}^{i}\left(r_{ib}^{i}\left(t\right)\right)dt=\sum_{j=1}^{m}\int_{t_{j-1}}^{t_{j}}g_{ib}^{i}\left(r_{ib}^{i}\left(t\right)\right)dt$$

$r_{ib}^{i}\left(t\right)$ can be obtained by the piecewise cubic spline interpolation in the time period $\left[t_{j-1},t_{j}\right]$, $T_{j}=t_{j}-t_{j-1}$.

(14) $$\begin{array}{c} r_{ib}^{i}\left(t\right)=\left\{T_{j}^{-2}\left[v_{ib}^{i}\left(t_{j}\right)+v_{ib}^{i}\left(t_{j-1}\right)\right]-2T_{j}^{-3}\left[r_{ib}^{i}\left(t_{j}\right)-r_{ib}^{i}\left(t_{j-1}\right)\right]\right\}{\left(t-t_{j-1}\right)}^{3}\\ +\left\{3T_{j}^{-2}\left[r_{ib}^{i}\left(t_{j}\right)-r_{ib}^{i}\left(t_{j-1}\right)\right]-T_{j}^{-1}\left[v_{ib}^{i}\left(t_{j}\right)+v_{ib}^{i}\left(t_{j-1}\right)\right]\right\}{\left(t-t_{j-1}\right)}^{2}+v_{ib}^{i}\left(t_{j-1}\right)\left(t-t_{j-1}\right)+r_{ib}^{i}\left(t_{j-1}\right) \end{array}$$

Differentiate Equation (14) twice, and let j=m, $\;t=t_{m}$ and $T_{m}=t_{m}-t_{m-1}$. ${\dot{v}}_{ib}^{i}\left(t_{m}\right)$ in Equation (12) can be calculated as

(15) $$\begin{array}{c} {\dot{v}}_{ib}^{i}\left(t_{m}\right)=6\left\{T_{m}^{-2}\left[v_{ib}^{i}\left(t_{m}\right)+v_{ib}^{i}\left(t_{m-1}\right)\right]-2T_{m}^{-3}\left[r_{ib}^{i}\left(t_{m}\right)-r_{ib}^{i}\left(t_{m-1}\right)\right]\right\}\left(t-t_{m-1}\right)\\ +2\left\{3T_{m}^{-2}\left[r_{ib}^{i}\left(t_{m}\right)-r_{ib}^{i}\left(t_{m-1}\right)\right]-T_{m}^{-1}\left[v_{ib}^{i}\left(t_{m}\right)+v_{ib}^{i}\left(t_{m-1}\right)\right]\right\} \end{array}$$

$\dot{q}\left(t_{m}\right)$ in Equation (12) can be obtained by the interpolation.

The piecewise cubic spline interpolation for the quaternion is expressed by [23]

(16) $$\bar{q}\left(t\right)={\bar{a}}_{m}t^{3}+{\bar{b}}_{m}t^{2}+{\bar{c}}_{m}t+{\bar{d}}_{m},\;t\in \left[t_{m-1},t_{m}\right],m\in \left\{1,2,3,\ldots M\right\}$$

(17) $$\dot{\bar{q}}\left(t\right)=3{\bar{a}}_{m}t^{2}+2{\bar{b}}_{m}t+{\bar{c}}_{m},\;t\in \left[t_{m-1},t_{m}\right],m\in \left\{1,2,3,\ldots M\right\}$$

where ${\bar{a}}_{m},{\bar{b}}_{m},{\bar{c}}_{m},{\bar{d}}_{m}$ are the coefficients of the polynomial, and they vary across the interpolation intervals. The subscript *m* (or m−1 in the following content) represents the time point.

$\bar{q}\left(t_{m}\right)$ represents the piecewise cubic spline quaternion in the time interval $t\in \left[t_{m-1},t_{m}\right]$

(18) $$\bar{q}\left(t_{m}\right)={\bar{a}}_{m}t_{m}^{3}+{\bar{b}}_{m}t_{m}^{2}+{\bar{c}}_{m}t_{m}+{\bar{d}}_{m}={\bar{a}}_{m-1}t_{m}^{3}+{\bar{b}}_{m-1}t_{m}^{2}+{\bar{c}}_{m-1}t_{m}+{\bar{d}}_{m-1}$$

(19) $$\dot{\bar{q}}\left(t_{m}\right)=3{\bar{a}}_{m}t_{m}^{2}+2{\bar{b}}_{m}t_{m}^{}+{\bar{c}}_{m}=3{\bar{a}}_{m-1}t_{m}^{2}+2{\bar{b}}_{m-1}t_{m}^{}+{\bar{c}}_{m-1}$$

(20) $$\ddot{\bar{q}}\left(t_{m}\right)=6{\bar{a}}_{m}t_{m}^{}+2{\bar{b}}_{m}=6{\bar{a}}_{m-1}t_{m}^{}+2{\bar{b}}_{m-1}$$

Considering the constraint condition $∥q\left(t\right)∥=1$, the quaternion interpolation and its derivatives can be rectified as follows:

(21a) $$q\left(t\right)=\frac{\bar{q}\left(t\right)}{∥\bar{q}\left(t\right)∥}$$

(21b) $$\dot{q}\left(t\right)=\frac{d}{dt}\left(\frac{\bar{q}\left(t\right)}{∥\bar{q}\left(t\right)∥}\right)=\frac{\dot{\bar{q}}\left(t\right)\cdot ∥\bar{q}\left(t\right)∥-\bar{q}\left(t\right)\cdot \frac{d}{dt}\left(∥\bar{q}\left(t\right)∥\right)}{{∥\bar{q}\left(t\right)∥}^{2}}$$

where,

(22) $$\frac{d}{dt}\left(∥\bar{q}\left(t\right)∥\right)=\frac{{\bar{q}}_{0}\left(t\right){\dot{\bar{q}}}_{0}\left(t\right)+{\bar{q}}_{1}\left(t\right){\dot{\bar{q}}}_{1}\left(t\right)+{\bar{q}}_{2}\left(t\right){\dot{\bar{q}}}_{2}\left(t\right)+{\bar{q}}_{3}\left(t\right){\dot{\bar{q}}}_{3}\left(t\right)}{∥\bar{q}\left(t\right)∥}$$

Let $t=t_{m}$, considering $∥\bar{q}\left(t_{m}\right)∥=∥q\left(t_{m}\right)∥=1$

(23) $$\begin{array}{cc} \dot{q}\left(t_{m}\right) & \;=\;\frac{\dot{\bar{q}}\left(t_{m}\right)\cdot ∥\bar{q}\left(t_{m}\right)∥\;-\;\bar{q}\left(t_{m}\right)\cdot \;\left[\;{\bar{q}}_{0}\left(t_{m}\right){\dot{\bar{q}}}_{0}\left(t_{m}\right)\;+\;{\bar{q}}_{1}\left(t_{m}\right){\dot{\bar{q}}}_{1}\left(t_{m}\right)\;+\;{\bar{q}}_{2}\left(t_{m}\right){\dot{\bar{q}}}_{2}\left(t_{m}\right)\;+\;{\bar{q}}_{3}\left(t_{m}\right){\dot{\bar{q}}}_{3}\left(t_{m}\right)\;\right]\;\;/\;∥\bar{q}\left(t_{m}\right)∥}{{∥\bar{q}\left(t_{m}\right)∥}^{2}}\\ & \;=\;\dot{\bar{q}}\left(t_{m}\right)-\bar{q}\left(t_{m}\right)\cdot \left[{\bar{q}}_{0}\left(t_{m}\right){\dot{\bar{q}}}_{0}\left(t_{m}\right)+{\bar{q}}_{1}\left(t_{m}\right){\dot{\bar{q}}}_{1}\left(t_{m}\right)+{\bar{q}}_{2}\left(t_{m}\right){\dot{\bar{q}}}_{2}\left(t_{m}\right)+{\bar{q}}_{3}\left(t_{m}\right){\dot{\bar{q}}}_{3}\left(t_{m}\right)\right] \end{array}$$

where $q_{0},q_{1},q_{2},q_{3}$ are the four elements of the quaternion, and ${\dot{q}}_{0},{\dot{q}}_{1},{\dot{q}}_{2},{\dot{q}}_{3}$ are the derivatives of the four elements of quaternion.

### 4.2. Two-Point Piecewise Cubic Spline Interpolation for Dual Quaternions

In order to obtain the analytic integrations of Equations (8) and (9), the cubic spline interpolation for the dual quaternion is expressed by

(24) $$\begin{array}{cc} \tilde{\overset{⌢}{\;q}}\left(t\right) & =\tilde{\overset{⌢}{q}}(t_{m-1}+\tau )=\overset{⌢}{q}(t_{m-1}+\tau )+\varepsilon \overset{⌢}{p}(t_{m-1}+\tau )\\ & =a(t_{m})\tau^{3}+b(t_{m})\tau^{2}+\dot{q}(t_{m})\tau +q(t_{m})+\varepsilon [c(t_{m})\tau^{3}+d(t_{m})\tau^{2}+\dot{p}(t_{m})\tau +p(t_{m})] \end{array}$$

(25) $$\begin{array}{cc} \dot{\tilde{\overset{⌢}{q}}}\left(t\right) & =\dot{\tilde{\overset{⌢}{\;q}}}(t_{m-1}+\tau )=\dot{\tilde{q}}(t_{m-1}+\tau )+\varepsilon \dot{\tilde{p}}(t_{m-1}+\tau )\\ & =3a(t_{m})\tau_{3}+2b(t_{m})\tau +\dot{q}(t_{m})+\varepsilon \left[3c(t_{m})\tau^{2}+2d(t_{m})\tau +\dot{p}(t_{m})\right] \end{array}$$

where, $T_{m}=t_{m}-t_{m-1}$, $\tau \in \left[0,T_{m}\right]$

(26) $$a\left(t_{m}\right)=\left[2T_{m}^{-3}\left(q\left(t_{m-1}\right)-q\left(t_{m}\right)\right)+T_{m}^{-2}\left(\dot{q}\left(t_{m-1}\right)+\dot{q}\left(t_{m}\right)\right)\right]$$

(27) $$b\left(t_{m}\right)=\left[-3T_{m}^{-2}\left(q\left(t_{m-1}\right)-q\left(t_{m}\right)\right)-T_{m}^{-1}\left(\dot{q}\left(t_{m-1}\right)+\dot{q}\left(t_{m}\right)\right)\right]$$

(28) $$c\left(t_{m}\right)=\left[2T_{m}^{-3}\left(p\left(t_{m-1}\right)-p\left(t_{m}\right)\right)+T_{m}^{-2}\left(\dot{p}\left(t_{m-1}\right)+\dot{p}\left(t_{m}\right)\right)\right]$$

(29) $$d\left(t_{m}\right)=\left[-3T_{m}^{-2}\left(p\left(t_{m-1}\right)-p\left(t_{m}\right)\right)-T_{m}^{-1}\left(\dot{p}\left(t_{m-1}\right)+\dot{p}\left(t_{m}\right)\right)\right]$$

subject to the following constraints of two-point boundary conditions

(30) $$\left\{\begin{array}{c} \tilde{\overset{⌢}{q}}\left(t_{m}\right)=\tilde{q}\left(t_{m}\right)+\varepsilon \tilde{p}\left(t_{m}\right)=q\left(t_{m}\right)+\varepsilon p\left(t_{m}\right)\\ \tilde{\overset{⌢}{\;q}}\left(t_{m-1}\right)=\tilde{q}\left(t_{m-1}\right)+\varepsilon \tilde{p}\left(t_{m-1}\right)=q\left(t_{m-1}\right)+\varepsilon p\left(t_{m-1}\right) \end{array}\right.$$

(31) $$\left\{\begin{array}{c} \dot{\tilde{\overset{⌢}{\;q}}}\left(t_{m}\right)=\dot{\tilde{q}}\left(t_{m}\right)+\varepsilon \dot{\tilde{p}}\left(t_{m}\right)=\dot{q}\left(t_{m}\right)+\varepsilon \dot{p}\left(t_{m}\right)\\ \dot{\tilde{\overset{⌢}{q}}}\left(t_{m-1}\right)=\dot{\tilde{q}}\left(t_{m-1}\right)+\varepsilon \dot{\tilde{p}}\left(t_{m-1}\right)=\dot{q}\left(t_{m-1}\right)+\varepsilon \dot{p}\left(t_{m-1}\right) \end{array}\right.$$

$\tilde{\overset{⌢}{\;q}}$ is the dual quaternion interpolation in the time interval *τ* ∈ [0, *T*_*m*_] without normalization, $\tilde{q}$ is the quaternion interpolation in the time interval *τ* ∈ [0, *T*_*m*_] without normalization, and $\tilde{p}$ is the dual part interpolation of $\tilde{\overset{⌢}{\;q}}$ in the time interval *τ* ∈ [0, *T*_*m*_].

### 4.3. Analytic Integration of Dual Quaternions

The twist, which is defined in the dual quaternion interpolations, is expressed by

(32) $${\tilde{\overset{⌢}{\;\omega }}}_{ib}^{b}=2{\tilde{\overset{⌢}{q}}}^{*}\circ \dot{\tilde{\overset{⌢}{q}}}={\overset{˜}{\omega }}_{ib}^{b}+\varepsilon {\overset{˜}{f}}_{ib}^{b}=2{\left(\overset{˜}{q}+\varepsilon \overset{˜}{p}\right)}^{*}\circ (\dot{\tilde{q}}+\varepsilon \dot{\tilde{p}})=2{\tilde{q}}^{*}\circ \dot{\tilde{q}}+\varepsilon 2({\tilde{q}}^{*}\circ +\dot{\tilde{p}}+{\tilde{p}}^{*}\circ \dot{\tilde{q}})$$

By substituting Equations (24) and (25) into Equation (32), integrating Equation (32) over the sampling time interval $\left[t_{k}-\Delta T,t_{k}\right]$ and supposing $t_{k}=t_{m-1}+\tau_{l}$, the following equations can be determined. 1/ΔT represents the integration frequency.

(33) $$\begin{array}{cc} \Delta \tilde{\theta }\left(t_{k}\right) & =\int_{t_{k}-\Delta T}^{t_{k}}{\tilde{\omega }}_{ib}^{b}\left(t\right)dt=2\int_{t_{k}-\Delta T}^{t_{k}}\left[{\tilde{q}}^{*}\left(t\right)\circ \dot{\tilde{q}}\left(t\right)\right]dt=2\int_{\tau_{l}-\Delta T}^{\tau_{l}}\left[{\tilde{q}}^{*}\left(t_{m-1}+\tau \right)\circ \dot{\tilde{q}}\left(t_{m-1}+\tau \right)\right]d\tau \\ & ={\left.-{\tilde{a}}^{*}\left(t_{m}\right)\circ \tilde{a}\left(t_{m}\right)\tau^{6}\right|}_{\tau_{l}-\Delta T}^{\tau_{l}}-\left[\frac{6}{5}a^{*}\left(t_{m}\right)\circ b\left(t_{m}\right)+\frac{4}{5}b^{*}\left(t_{m}\right)\circ a\left(t_{m}\right)\right]{\left.\tau^{5}\right|}_{\tau_{l}-\Delta T}^{\tau_{l}}\\ & -{\left.\left[\frac{3}{2}a^{*}\left(t_{m}\right)\circ \dot{q}\left(t_{m}\right)+b^{*}\left(t_{m}\right)\circ b\left(t_{m}\right)+\frac{1}{2}{\dot{q}}^{*}\left(t_{m}\right)\circ a\left(t_{m}\right)\right]\tau^{4}\right|}_{\tau_{l}-\Delta T}^{\tau_{l}}\\ & -{\left.\left[2a^{*}\left(t_{m}\right)\circ q\left(t_{m}\right)+\frac{4}{3}b^{*}\left(t_{m}\right)\circ \dot{q}\left(t_{m}\right)+\frac{2}{3}{\dot{q}}^{*}\left(t_{m}\right)\circ b\left(t_{m}\right)\right]\tau^{3}\right|}_{\tau_{l}-\Delta T}^{\tau_{l}}\\ & -{\left.\left[2b^{*}\left(t_{m}\right)\circ q\left(t_{m}\right)+{\dot{q}}^{*}\left(t_{m}\right)\circ \dot{q}\left(t_{m}\right)\right]\tau^{2}\right|}_{\tau_{l}-\Delta T}^{\tau_{l}}-2{\dot{q}}^{*}\left(t_{m}\right)\circ q\left(t_{m}\right){\left.\tau \right|}_{\tau_{l}-\Delta T}^{\tau_{l}} \end{array}$$

(34) $$\begin{array}{cc} \Delta \tilde{\upsilon }\left(t_{k}\right) & =\int_{t_{k}-\Delta T}^{t_{k}}{\tilde{f}}_{ib}^{b}\left(t\right)dt={\left.2{\tilde{q}}^{*}\left(t\right)\circ \tilde{p}\left(t\right)\right|}_{t_{k}-\Delta T}^{t_{k}}-4\int_{t_{k}-\Delta T}^{t_{k}}\left[{\dot{\tilde{q}}}^{*}\left(t\right)\circ \tilde{p}\left(t\right)\right]dt\\ & ={\left.2{\tilde{q}}^{*}\left(t_{m-1}+\tau \right)\circ \tilde{p}\left(t_{m-1}+\tau \right)\right|}_{\tau_{l}-\Delta T}^{\tau_{l}}-4\int_{\tau_{l}-\Delta T}^{\tau_{l}}\left[{\dot{\tilde{q}}}^{*}\left(t_{m-1}+\tau \right)\circ \tilde{p}\left(t_{m-1}+\tau \right)\right]d\tau \\ & =2{\left.a^{*}\left(t_{m}\right)\circ c\left(t_{m}\right)\tau^{6}\right|}_{\tau_{l}-\Delta T}^{\tau_{l}}+\left[2a^{*}\left(t_{m}\right)\circ d\left(t_{m}\right)+2b^{*}\left(t_{m}\right)\circ c\left(t_{m}\right)\right]{\left.\tau^{5}\right|}_{\tau_{l}-\Delta T}^{\tau_{l}}\\ & +{\left.\left[2a^{*}\left(t_{m}\right)\circ \dot{p}\left(t_{m}\right)+2b^{*}\left(t_{m}\right)\circ d\left(t_{m}\right)+2{\dot{q}}^{*}\left(t_{m}\right)\circ c\left(t_{m}\right)\right]\tau^{4}\right|}_{\tau_{l}-\Delta T}^{\tau_{l}}\\ & +{\left.\left[2a^{*}\left(t_{m}\right)\circ p\left(t_{m}\right)+2b^{*}\left(t_{m}\right)\circ \dot{p}\left(t_{m}\right)+2{\dot{q}}^{*}\left(t_{m}\right)\circ d\left(t_{m}\right)+2q^{*}\left(t_{m}\right)\circ c\left(t_{m}\right)\right]\tau^{3}\right|}_{\tau_{l}-\Delta T}^{\tau_{l}}\\ & +{\left.\left[2b^{*}\left(t_{m}\right)\circ p\left(t_{m}\right)+2{\dot{q}}^{*}\left(t_{m}\right)\circ \dot{p}\left(t_{m}\right)+2q^{*}\left(t_{m}\right)\circ d\left(t_{m}\right)\right]\tau^{2}\right|}_{\tau_{l}-\Delta T}^{\tau_{l}}\\ & +{\left.\left[2{\dot{q}}^{*}\left(t_{m}\right)\circ p\left(t_{m}\right)+2q^{*}\left(t_{m}\right)\circ \dot{p}\left(t_{m}\right)\right]\tau \right|}_{\tau_{l}-\Delta T}^{\tau_{l}}+2q^{*}\left(t_{m}\right)\circ p\left(t_{m}\right)\\ & -{\left.2a^{*}\left(t_{m}\right)\circ c\left(t_{m}\right)\tau^{6}\right|}_{\tau_{l}-\Delta T}^{\tau_{l}}-\left[\frac{12}{5}a^{*}\left(t_{m}\right)\circ d\left(t_{m}\right)+\frac{8}{5}b^{*}\left(t_{m}\right)\circ c\left(t_{m}\right)\right]{\left.\tau^{5}\right|}_{\tau_{l}-\Delta T}^{\tau_{l}}\\ & -{\left.\left[3a^{*}\left(t_{m}\right)\circ \dot{p}\left(t_{m}\right)+2b^{*}\left(t_{m}\right)\circ d\left(t_{m}\right)+{\dot{q}}^{*}\left(t_{m}\right)\circ c\left(t_{m}\right)\right]\tau^{4}\right|}_{\tau_{l}-\Delta T}^{\tau_{l}}\\ & -{\left.\left[4a^{*}\left(t_{m}\right)\circ p\left(t_{m}\right)+\frac{8}{3}b^{*}\left(t_{m}\right)\circ \dot{p}\left(t_{m}\right)+\frac{4}{3}{\dot{q}}^{*}\left(t_{m}\right)\circ d\left(t_{m}\right)\right]\tau^{3}\right|}_{\tau_{l}-\Delta T}^{\tau_{l}}\\ & -{\left.\left[4b^{*}\left(t_{m}\right)\circ p\left(t_{m}\right)+2{\dot{q}}^{*}\left(t_{m}\right)\circ \dot{p}\left(t_{m}\right)\right]\tau^{2}\right|}_{\tau_{l}-\Delta T}^{\tau_{l}}-4{\dot{q}}^{*}\left(t_{m}\right)\circ p\left(t_{m}\right){\left.\tau \right|}_{\tau_{l}-\Delta T}^{\tau_{l}} \end{array}$$

### 4.4. Norm Corrections of Dual Quaternion

The norm of the dual quaternion representing rotation and translation between the inertial frame and the body frame is defined as

(35) $$\begin{array}{cc} {∥\overset{⌢}{q}∥}^{2} & =\overset{⌢}{\;q}\circ {\overset{⌢}{q}}^{*}=(q+\varepsilon p)\circ {\left(q+\varepsilon p\right)}^{*}=(q+\varepsilon p)\circ (q^{*}+\varepsilon p^{*})=q\circ q^{*}+\varepsilon (q\circ p^{*}+p\circ q^{*})\\ & =q\circ q^{*}+\varepsilon (q\circ {\left(\frac{1}{2}t^{i}\circ q\right)}^{*}+(\frac{1}{2}t^{i}\circ q)\circ q^{*})\\ & =1+\varepsilon (q\circ q^{*}\circ (-\frac{1}{2}t^{i})+t^{i}\circ q\circ q^{*})=1 \end{array}$$

in which, $q\circ q^{*}=q_{0}^{2}+q_{1}^{2}+q_{2}^{2}+q_{3}^{2}=1$ , $q\circ p^{*}+p\circ q^{*}=2\left(q_{0}p_{0}+q_{1}p_{1}+q_{2}p_{2}+q_{3}p_{3}\right)=0$ and ${∥\overset{⌢}{\;q}∥}^{2}$ is the square of the dual quaternion norm. Generally, the cubic spline interpolation $\tilde{q}\left(t\right)$ and $\tilde{p}\left(t\right)$ do not satisfy these constraints, which results in undesirable fluctuation errors in $\Delta \tilde{\theta }\left(t_{k}\right)$ and $\Delta \tilde{\upsilon }\left(t_{k}\right)$.

To solve these problems, norm corrections of the dual quaternion interpolation is brought into the analytic integrations as follows:

(36a) $$\overset{⌢}{\;q}=\tilde{\overset{⌢}{q}}/∥\tilde{\overset{⌢}{q}}∥$$

(36b) $$\dot{\overset{⌢}{\;q}}=\left(\dot{\tilde{\overset{⌢}{q}}}∥\tilde{\overset{⌢}{q}}∥-\tilde{\overset{⌢}{q}}∥\dot{\tilde{\overset{⌢}{q}}}∥\right)/{∥\tilde{\overset{⌢}{q}}∥}^{2}$$

Substituting Equation (36) into Equation (7), the corrected twist is derived as

(37) $$\begin{array}{cc} {\overset{⌢}{\omega }}_{ib}^{b} & =\omega_{ib}^{b}+\varepsilon f_{ib}^{b}\\ & =2\frac{{\tilde{\overset{⌢}{q}}}^{*}}{\tilde{\overset{⌢}{q}}}\circ \frac{\dot{\tilde{\overset{⌢}{q}}}\circ ∥\tilde{\overset{⌢}{q}}∥-\tilde{\overset{⌢}{q}}\circ \frac{d}{dt}∥\tilde{\overset{⌢}{q}}∥}{{∥\tilde{\overset{⌢}{q}}∥}^{2}}=\frac{2{\tilde{\overset{⌢}{q}}}^{*}\circ \dot{\tilde{\overset{⌢}{q}}}}{{∥\tilde{\overset{⌢}{q}}∥}^{2}}-\frac{2\frac{d}{dt}∥\tilde{\overset{⌢}{q}}∥}{∥\tilde{\overset{⌢}{q}}∥}\\ & =\frac{{\tilde{\overset{⌢}{\omega }}}_{ib}^{b}}{{∥\tilde{\overset{⌢}{q}}∥}^{2}}-\frac{2\frac{d}{dt}∥\tilde{\overset{⌢}{q}}∥}{∥\tilde{\overset{⌢}{q}}∥}=\frac{{\overset{˜}{\omega }}_{ib}^{b}+\varepsilon {\overset{˜}{f}}_{ib}^{b}}{{∥\tilde{\overset{⌢}{q}}∥}^{2}}-\frac{2\frac{d}{dt}∥\tilde{\overset{⌢}{q}}∥}{∥\tilde{\overset{⌢}{q}}∥} \end{array}$$

where

(38) $$\begin{array}{cc} \frac{1}{{∥\tilde{\overset{⌢}{\;q}}∥}^{2}} & =\frac{1}{\left(\tilde{q}+\varepsilon \tilde{p}\right)\circ {\left(\tilde{q}+\varepsilon \tilde{p}\right)}^{*}}=\frac{1}{\tilde{q}\circ {\tilde{q}}^{*}+\varepsilon \left(\tilde{q}\circ {\tilde{p}}^{*}+\tilde{p}\circ {\tilde{q}}^{*}\right)}\\ & =\frac{1}{{∥\tilde{q}∥}^{2}+\varepsilon \left(\tilde{q}\circ {\tilde{p}}^{*}+\tilde{p}\circ {\tilde{q}}^{*}\right)}\cdot \frac{{∥\tilde{q}∥}^{2}-\varepsilon \left(\tilde{q}\circ {\tilde{p}}^{*}+\tilde{p}\circ {\tilde{q}}^{*}\right)}{{∥\tilde{q}∥}^{2}-\varepsilon \left(\tilde{q}\circ {\tilde{p}}^{*}+\tilde{p}\circ {\tilde{q}}^{*}\right)}=\frac{{∥\tilde{q}∥}^{2}-\varepsilon \left(\tilde{q}\circ {\tilde{p}}^{*}+\tilde{p}\circ {\tilde{q}}^{*}\right)}{{∥\tilde{q}∥}^{4}}\\ & =\frac{1}{{∥\tilde{q}∥}^{2}}-2\varepsilon \frac{{\tilde{q}}_{0}{\tilde{p}}_{0}+{\tilde{q}}_{1}{\tilde{p}}_{1}+{\tilde{q}}_{2}{\tilde{p}}_{2}+{\tilde{q}}_{3}{\tilde{p}}_{3}}{{∥\tilde{q}∥}^{4}} \end{array}$$

Substituting Equation (38) into Equation (37), the angular rates and specific force can be expressed as

(39) $$\omega_{ib}^{b}+\varepsilon f_{ib}^{b}=\frac{{\tilde{\omega }}_{ib}^{b}}{{∥\tilde{q}∥}^{2}}+\varepsilon \left[\frac{{\tilde{f}}_{ib}^{b}}{{∥\tilde{q}∥}^{2}}-\frac{2\left({\tilde{q}}_{0}{\tilde{p}}_{0}+{\tilde{q}}_{1}{\tilde{p}}_{1}+{\tilde{q}}_{2}{\tilde{p}}_{2}+{\tilde{q}}_{3}{\tilde{p}}_{3}\right){\tilde{\omega }}_{ib}^{b}}{{∥\tilde{q}∥}^{4}}\right]-\frac{2\frac{d}{dt}∥\tilde{\overset{⌢}{\;q}}∥}{∥\tilde{\overset{⌢}{q}}∥}$$

$\frac{2\frac{d}{dt}∥\tilde{\overset{⌢}{q}}∥}{∥\tilde{\overset{⌢}{\;q}}}$ is scalar and can be neglected in the vector computation for $\omega_{ib}^{b}$ and $f_{ib}^{b}$.

Considering the fact that sampling time ΔT is generally marginal (5 ms or less) over the sampling time interval $\left[t_{k}-\Delta T,t_{k}\right]$, the following approximations can be adopted:

(40) $$\frac{1}{{∥\tilde{q}∥}^{2}}\approx \frac{2}{{∥\tilde{q}\left(t_{k}-\Delta T\right)∥}^{2}+{∥\tilde{q}\left(t_{k}\right)∥}^{2}}$$

(41) $$\begin{array}{c} 2\frac{\left({\tilde{q}}_{0}{\tilde{p}}_{0}+{\tilde{q}}_{1}{\tilde{p}}_{1}+{\tilde{q}}_{2}{\tilde{p}}_{2}+{\tilde{q}}_{3}{\tilde{p}}_{3}\right)}{{∥\tilde{q}∥}^{4}}\approx A\left(t_{k}\right)\\ \;\approx \;\frac{{\tilde{q}}_{0}\left(t_{k}\;-\;\Delta T\right){\tilde{p}}_{0}\left(t_{k}\;-\;\Delta T\right)\;+\;{\tilde{q}}_{1}\left(t_{k}\;-\;\Delta T\right){\tilde{p}}_{1}\left(t_{k}\;-\;\Delta T\right)}{{∥\tilde{q}\left(t_{k}\;-\;\Delta T\right)∥}^{4}}\;+\;\frac{{\tilde{q}}_{2}\left(t_{k}\;-\;\Delta T\right){\tilde{p}}_{2}\left(t_{k}\;-\;\Delta T\right)\;+\;{\tilde{q}}_{3}\left(t_{k}\;-\;\Delta T\right){\tilde{p}}_{3}\left(t_{k}\;-\;\Delta T\right)}{{∥\tilde{q}\left(t_{k}\;-\;\Delta T\right)∥}^{4}}\\ +\frac{{\tilde{q}}_{0}\left(t_{k}\right){\tilde{p}}_{0}\left(t_{k}\right)+{\tilde{q}}_{1}\left(t_{k}\right){\tilde{p}}_{1}\left(t_{k}\right)+{\tilde{q}}_{2}\left(t_{k}\right){\tilde{p}}_{2}\left(t_{k}\right)+{\tilde{q}}_{3}\left(t_{k}\right){\tilde{p}}_{3}\left(t_{k}\right)}{{∥\tilde{q}\left(t_{k}\right)∥}^{4}} \end{array}$$

Based on the above equations, the integral angular rate increments in Equation (8) and specific force integral increments in Equation (9) can be rewritten as

(42) $$\begin{array}{cc} \Delta \theta \left(t_{k}\right) & =\int_{t_{k}-\Delta T}^{t_{k}}\omega_{ib}^{b}\left(t\right)dt\approx \frac{2}{{∥\tilde{q}\left(t_{k}-\Delta T\right)∥}^{2}+{∥\tilde{q}\left(t_{k}\right)∥}^{2}}\int_{t_{k}-\Delta T}^{t_{k}}{\tilde{\omega }}_{ib}^{b}\left(t\right)dt\\ & =\frac{2}{{∥\tilde{q}\left(t_{k}-\Delta T\right)∥}^{2}+{∥\tilde{q}\left(t_{k}\right)∥}^{2}}\cdot \Delta \tilde{\theta }\left(t_{k}\right) \end{array}$$

(43) $$\begin{array}{cc} \Delta \upsilon \left(t_{k}\right) & =\int_{t_{k}-\Delta T}^{t_{k}}f_{ib}^{b}\left(t\right)dt\approx \frac{2}{{∥\tilde{q}\left(t_{k}-\Delta T\right)∥}^{2}+{∥\tilde{q}\left(t_{k}\right)∥}^{2}}\int_{t_{k}-\Delta T}^{t_{k}}{\tilde{f}}_{ib}^{b}\left(t\right)dt-A\left(t_{k}\right)\int_{t_{k}-\Delta T}^{t_{k}}{\tilde{\omega }}_{ib}^{b}\left(t\right)dt\\ & =\frac{2}{{∥\tilde{q}\left(t_{k}-\Delta T\right)∥}^{2}+{∥\tilde{q}\left(t_{k}\right)∥}^{2}}\Delta \tilde{\upsilon }\left(t_{k}\right)-A\left(t_{k}\right)\Delta \tilde{\theta }\left(t_{k}\right) \end{array}$$

By this procedure, simulated IMU signals including gyro measurements and accelerometer measurements can be calculated by the dual quaternion interpolation and the analytic integration.

As $\omega_{ib}^{b}$ and ${\dot{\omega }}_{ib}^{b}$ can be obtained analytically, the error sources such as the size effects and the lever arm effects can also be simulated [24]. For example, when $r_{bx}^{b}$, $r_{by}^{b}$ and $r_{bz}^{b}$ (the displacements of the x-, y- and z- axis accelerometers, respectively, from the IMU reference point expressed in frame *b* ) are specified, the size effect caused by the displacements of the accelerometers from the IMU reference point can be simulated. Similarly, when $r_{ba}^{b}$ (the displacement of the GNSS receiver antenna from the IMU reference point expressed in frame *b* ) is specified, the lever arm effect caused by the displacement of the GNSS receiver antenna from the IMU reference point can also be simulated.

### 4.5. Error Analysis and Methods for Accuracy Enhancement

The error caused by approximations in Equations (40) and (41) can be expressed as:

(44) $$\delta \omega_{ib}^{b}=\left(\frac{1}{{∥\tilde{q}∥}^{2}}-\frac{2}{{∥\tilde{q}\left(t_{k}-\Delta T\right)∥}^{2}+{∥\tilde{q}\left(t_{k}\right)∥}^{2}}\right)\omega_{ib}^{b}$$

(45) $$\delta f_{ib}^{b}=\left(\frac{1}{{∥\tilde{q}∥}^{2}}-\frac{2}{{∥\tilde{q}\left(t_{k}-\Delta T\right)∥}^{2}+{∥\tilde{q}\left(t_{k}\right)∥}^{2}}\right)f_{ib}^{b}-\left(\frac{2\left({\tilde{q}}_{0}{\tilde{p}}_{0}+{\tilde{q}}_{1}{\tilde{p}}_{1}+{\tilde{q}}_{2}{\tilde{p}}_{2}+{\tilde{q}}_{3}{\tilde{p}}_{3}\right)}{{∥\tilde{q}∥}^{4}}-A\left(t_{k}\right)\right)\omega_{ib}^{b}$$

where $A\left(t_{k}\right)$ is defined in Equation (41), $\delta \omega_{ib}^{b}$ is the error vector of the angular rate, and $\delta f_{ib}^{b}$ is the error vector of the specific force.

For a low dynamic vehicle trajectory, amplitudes of $\omega_{ib}^{b}$ and $f_{ib}^{b}$ are generally negligible; therefore, errors in Equations (44) and (45) can be neglected. For a high dynamic vehicle trajectory with large angular rates and specific force, a smaller sampling integration time interval ΔT is to be selected to reduce the errors.

In terms of the computational complexity, this algorithm only needs a series of sequential calculations including interpolation, differentiation, multiplication and integration for each point. When there are *n* points, the number of calculation steps is proportional to *n*. Therefore, the computational complexity is $O\left(n\right)$.

## 5. Simulation Tests for Analytic IMU Signal Generator Performance Validation

This section describes the numerical simulations to validate the proposed algorithm. We choose the quaternion error and the position error as the test metrics to illustrate the performance of the algorithm. The quaternion error means the error between the quaternion of the original data we input and the quaternion of the results we obtained. The position error means the error between the position in ECI coordinates of the original data we input and the position in ECI coordinates of the results we obtained. The results of quaternion and the position in ECI coordinates are obtained by using the strapdown inertial navigation solution with the angular increment and the specific force increment obtained by the proposed algorithm.

**Figure 2:**
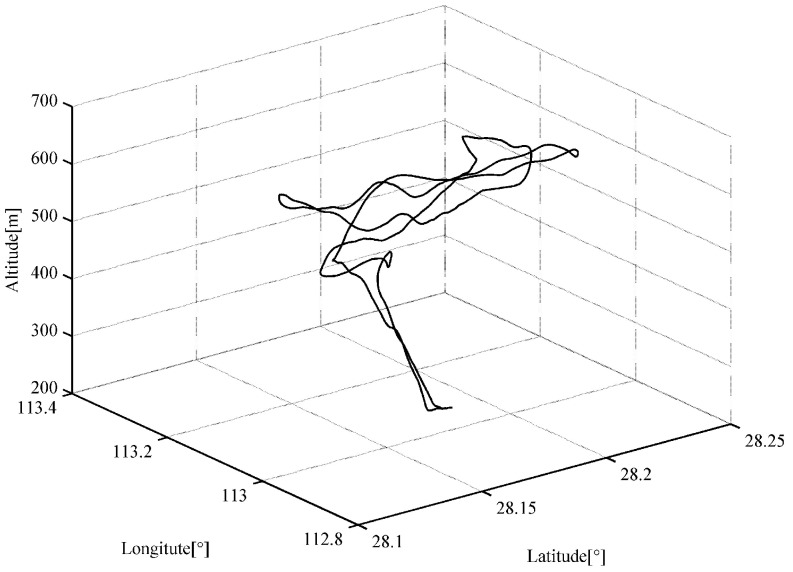
Actual flight trajectory.

The actual flight data including position, velocity, attitude and time as well as GNSS’s pseudoranges and their rate measurements have been procured from post-processed GNSS navigation solutions and a low-cost loosely coupled SINS/GNSS integrated navigation system in a UAV. The information of the attitude and the position are used for the algorithm proposed in this paper to generate the IMU signal. The update rate of flight data is 1 Hz and the total duration is 1200 s. The flight profile includes climbing, turning, cruise and a few flying manoeuvres, which are illustrated in Figure 2.

**Figure 3:**
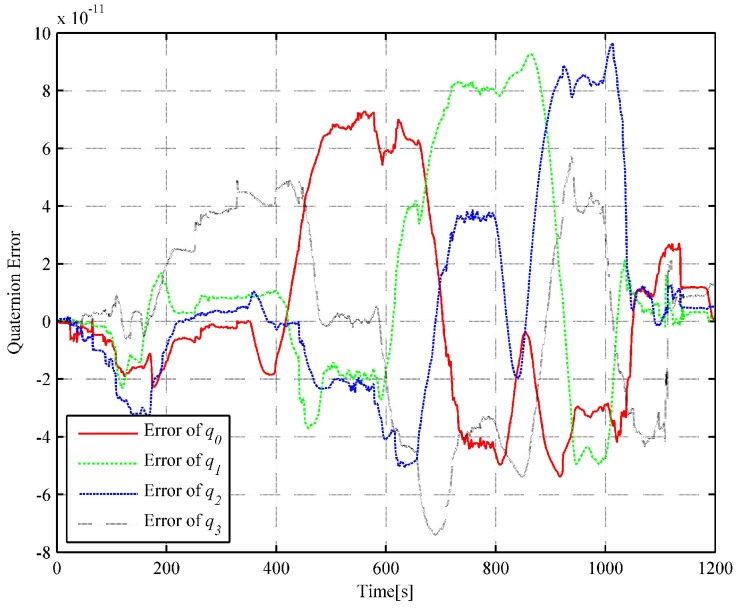
Curve of the quaternion error (based on DQ).

**Figure 4:**
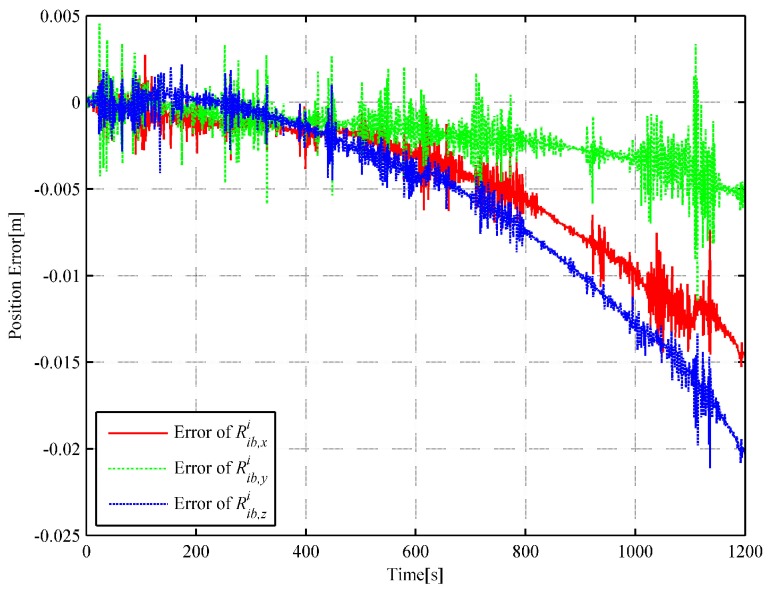
Curve of the position error (based on DQ).

We choose 1 s as the analytic interpolation interval, i.e., $T_{interp}=T=$1, which is consistent with the sampling frequency of the satellite signals. The simulated angular rate integral increments and the specific force integral increments are generated based on the dual quaternion interpolations at a frequency of 400 Hz. That is to say, ΔT=0.0025 s. It is a common sampling frequency of SINS in engineering. The strapdown inertial navigation simulation is performed to verify the accuracy of the simulated gyro and accelerometer measurements. The coning and sculling compensation algorithms are used for the attitude and velocity update, while the trapezoidal integration method is used for the position update [24]. The strapdown inertial navigation simulation results are compared with the flight trajectory at time $t_{m}$ without considering the initial alignment errors and instrument errors such as gyro drifts, accelerometer biases and scale factor errors. The attitude and position error comparisons of the simulation results are illustrated in Figure 3 and Figure 4, respectively.

It can be observed in Figure 3 and Figure 4 that the attitude quaternion error and position error accumulate over time, which is consistent with the inertial navigation’s error characteristics (the attitude error and the position error accumulate with time). In Figure 4, the fluctuation is mainly owing to the use of the average approximation and the computing error, and it can be neglected. The quaternion error is smaller than $9.66\times 10^{-11}$, and the position error is smaller than 0.021 m. The simulated angular rate increments and specific force integral increments are highly satisfactory and show high accuracy because the errors in Figure 3 and Figure 4 are substantially smaller than those caused by misalignments or bias and scale factor errors of gyros and accelerometers.

**Figure 5:**
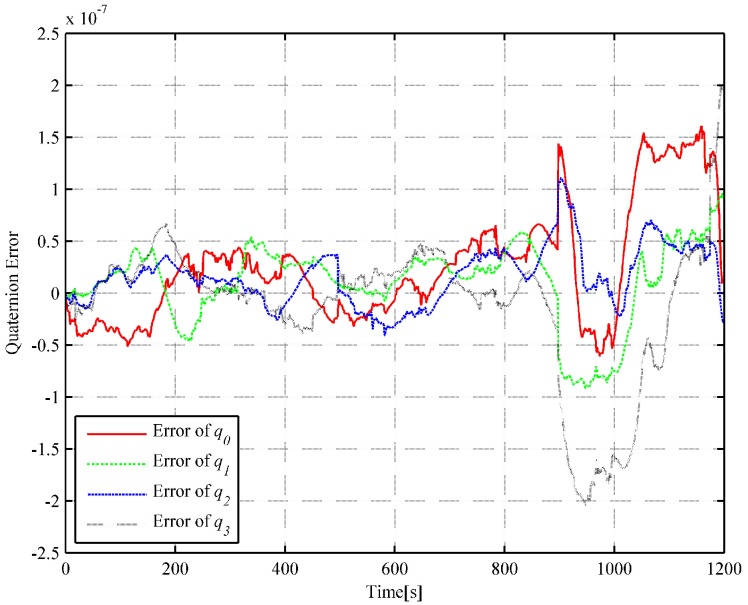
Curve of the quaternion error (based on the inverse SINS).

**Figure 6:**
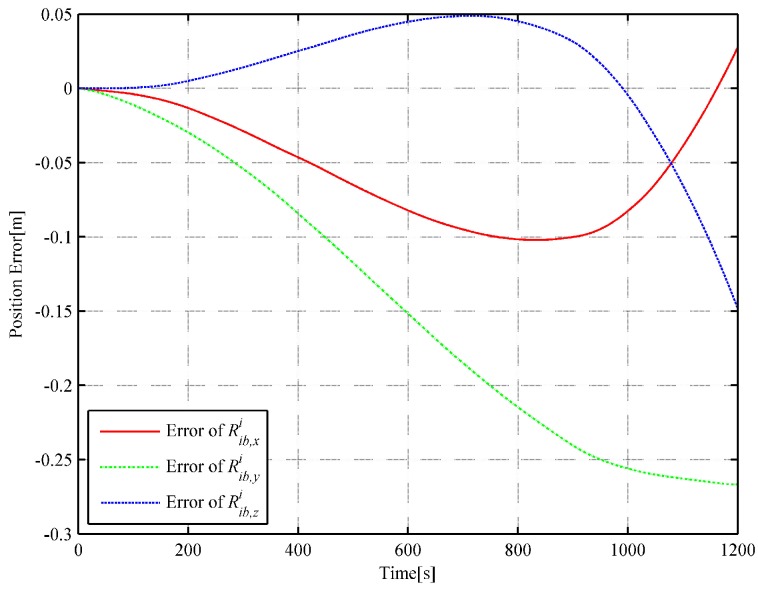
Curve of the position error (based on the inverse SINS).

In order to further illustrate the algorithm’s performance, the inverse SINS algorithm in [18] and the trajectory generation algorithm based on quaternion [19] are used with the same actual flight data. For the inverse SINS algorithm, the interpolation interval is also set as 1 s and the integrated frequency is 400 Hz. The attitude and position error comparisons of the simulation results are illustrated in Figure 5 and Figure 6, respectively.

According to the trajectory generation algorithm based on the quaternion [19], the following can be obtained:

(46) $$\omega_{ib}^{b}\left(t\right)=\frac{2}{{∥{\bar{q}}_{k}\left(t\right)∥}^{2}}\left[\begin{array}{cccc} -{\bar{q}}_{1,k}\left(t\right) & {\bar{q}}_{0,k}\left(t\right) & {\bar{q}}_{3,k}\left(t\right) & -{\bar{q}}_{2,k}\left(t\right)\\ -{\bar{q}}_{2,k}\left(t\right) & -{\bar{q}}_{3,k}\left(t\right) & {\bar{q}}_{0,k}\left(t\right) & {\bar{q}}_{1,k}\left(t\right)\\ -{\bar{q}}_{3,k}\left(t\right) & {\bar{q}}_{2,k}\left(t\right) & -{\bar{q}}_{1,k}\left(t\right) & {\bar{q}}_{0,k}\left(t\right) \end{array}\right]\left[\begin{array}{c} {\dot{\bar{q}}}_{0,k}\left(t\right)\\ {\dot{\bar{q}}}_{1,k}\left(t\right)\\ {\dot{\bar{q}}}_{2,k}\left(t\right)\\ {\dot{\bar{q}}}_{3,k}\left(t\right) \end{array}\right]$$

(47) $$\begin{array}{cc} f_{ib,k}^{b}\left(t\right) & =a_{ib,k}^{b}\left(t\right)-g_{ib,k}^{b}\left(t\right)\\ & =C_{i,k}^{b}\left(t\right)a_{ib,k}^{i}\left(t\right)-C_{i,k}^{b}\left(t\right)g_{ib,k}^{i}\left(t\right)\\ & =\frac{1}{{∥{\bar{q}}_{k}\left(t\right)∥}^{2}}\left[\begin{array}{ccc} {\bar{c}}_{11,k}\left(t\right) & {\bar{c}}_{21,k}\left(t\right) & {\bar{c}}_{31,k}\left(t\right)\\ {\bar{c}}_{12,k}\left(t\right) & {\bar{c}}_{22,k}\left(t\right) & {\bar{c}}_{32,k}\left(t\right)\\ {\bar{c}}_{13,k}\left(t\right) & {\bar{c}}_{23,k}\left(t\right) & {\bar{c}}_{33,k}\left(t\right) \end{array}\right]\left[a_{ib,k}^{i}\left(t\right)-g_{ib,k}^{i}\left(t\right)\right] \end{array}$$

where ${\bar{c}}_{ij,k}\left(t\right)$ is the function of quaternion ${\bar{q}}_{k}\left(t\right)$ . Using the first-order Taylor expansion for $1/{∥{\bar{q}}_{k}\left(t\right)∥}^{2}$, Equations (46) and (47) can be rewritten as

(48) $$\omega_{ib}^{b}\left(t\right)=\left(2-{∥{\bar{q}}_{k}\left(t\right)∥}^{2}\right)\left[\begin{array}{cccc} -{\bar{q}}_{1,k}\left(t\right) & {\bar{q}}_{0,k}\left(t\right) & {\bar{q}}_{3,k}\left(t\right) & -{\bar{q}}_{2,k}\left(t\right)\\ -{\bar{q}}_{2,k}\left(t\right) & -{\bar{q}}_{3,k}\left(t\right) & {\bar{q}}_{0,k}\left(t\right) & {\bar{q}}_{1,k}\left(t\right)\\ -{\bar{q}}_{3,k}\left(t\right) & {\bar{q}}_{2,k}\left(t\right) & -{\bar{q}}_{1,k}\left(t\right) & {\bar{q}}_{0,k}\left(t\right) \end{array}\right]\left[\begin{array}{c} {\dot{\bar{q}}}_{0,k}\left(t\right)\\ {\dot{\bar{q}}}_{1,k}\left(t\right)\\ {\dot{\bar{q}}}_{2,k}\left(t\right)\\ {\dot{\bar{q}}}_{3,k}\left(t\right) \end{array}\right]$$

(49) $$f_{ib,k}^{b}\left(t\right)=\left(2-{∥{\bar{q}}_{k}\left(t\right)∥}^{2}\right)\left[\begin{array}{ccc} {\bar{c}}_{11,k}\left(t\right) & {\bar{c}}_{21,k}\left(t\right) & {\bar{c}}_{31,k}\left(t\right)\\ {\bar{c}}_{12,k}\left(t\right) & {\bar{c}}_{22,k}\left(t\right) & {\bar{c}}_{32,k}\left(t\right)\\ {\bar{c}}_{13,k}\left(t\right) & {\bar{c}}_{23,k}\left(t\right) & {\bar{c}}_{33,k}\left(t\right) \end{array}\right]\left[a_{ib,k}^{i}\left(t\right)-g_{ib,k}^{i}\left(t\right)\right]$$

**Figure 7:**
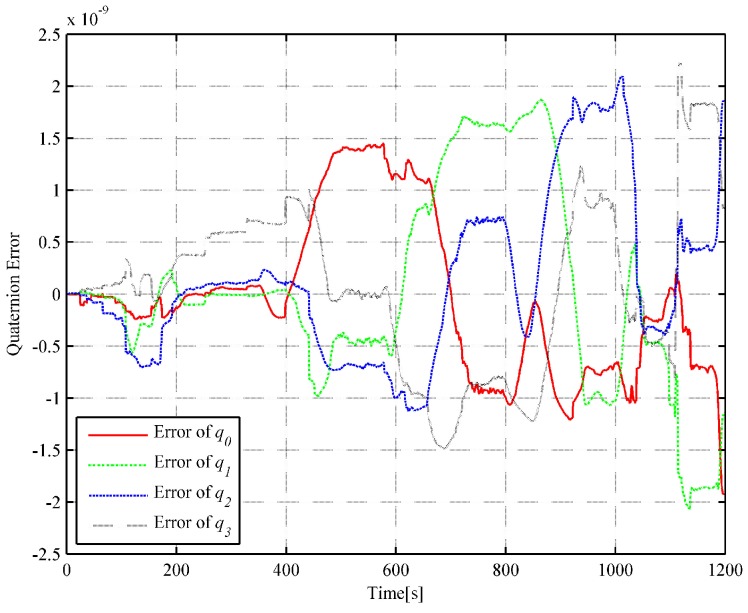
Curve of the quaternion error (based on the quaternion).

**Figure 8:**
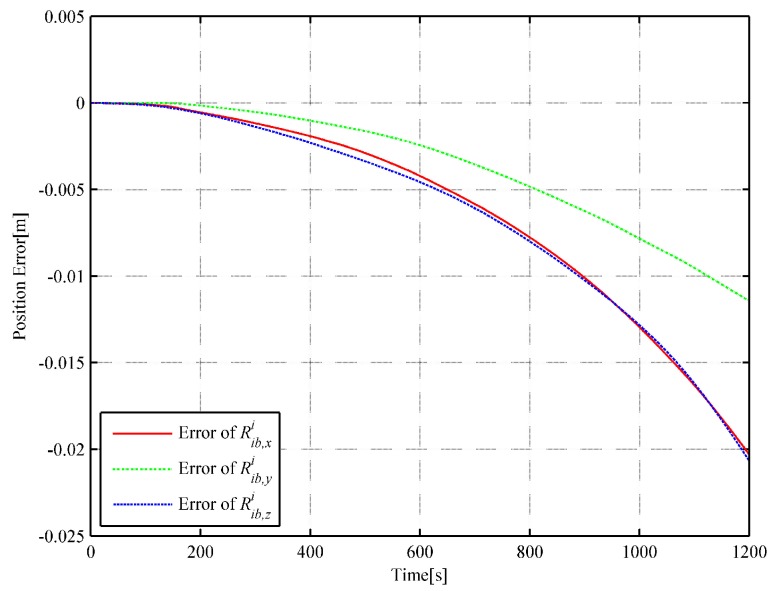
Curve of position error (based on quaternion).

With the same actual flight data, the error result can be obtained by MATLAB simulation. The attitude and the position errors are illustrated in Figure 7 and Figure 8, respectively.

In the strapdown inertial navigation system, the attitude accuracy is highly critical for the whole system’s calculation. Through the simulation, it can be observed that the dual quaternion interpolation algorithm developed in this study shows the highest attitude accuracy, with an error of $9.660722\times 10^{-11}$. The error in the quaternion interpolation algorithm $\left(2.225313\times 10^{-9}\right)$ is lower than that of the inverse SINS algorithm in [18]$\left(2.048823\times 10^{-7}\right)$. Then, the position errors of the three algorithms are compared. From Figure 4, Figure 6 and Figure 8, it can be observed that the dual quaternion interpolation algorithm developed in this study shows the highest accuracy also, with a position norm error of $2.575336\times 10^{-2}$ m. The quaternion interpolation algorithm is the second, with a position norm error of h m. The performance of the inverse SINS algorithm is the worst, with a position norm error of $3.063631\times 10^{-1}$ m. Furthermore, high order Taylor expansion approximation can be used in Equations (46) and (47) to enhance attitude and position accuracy and the algorithm’s performance. However, when comparing Equation (42) with Equation (48) and Equation (43) with Equation (49), it is observed that Equations (48) and (49) show higher order than Equations (42) and (43), although only the first-order Taylor expansion approximation is used. An increase in the order of the equations increases the time cost.

The computation time of the three algorithms is shown in Table 1. From the data in the table we can see that the computation time of the three algorithms is similar for the dataset containing 1200 data points. The algorithm based on the inverse SINS takes the most time and its accuracy is the worst among the three algorithms. Although the proposed algorithm takes slightly more time than the algorithm based on quaternion, the attitude accuracy obtained by the former one is better than that of the latter one. The longer time is acceptable.

Therefore, it can also be concluded that the analytic IMU signal generation algorithm, which is based on the actual flight data of a UAV and dual quaternion interpolations, show high accuracy. Moreover, it is capable of satisfying the requirements of dynamic simulations of SINS/GNSS integrated navigation research studies, in which simulated IMU signals are to be consistent with GNSS’s pseudoranges and their rate measurements in an actual flight test.

## 6. Conclusions

This paper proposed a new analytical IMU signal generation algorithm based on the dual quaternion interpolation. Compared with other algorithms, the new algorithm provides a concise analytical form for angular rate increments and specific force integral increments, and has higher accuracy than other state-of-the-art algorithms. The simulation results indicate that the quaternion error is smaller than $9.66\times 10^{-11}$, and the position error is smaller than 0.021 m. The accuracy of the proposed algorithm is capable of satisfying the requirements of dynamic simulations of the integrated navigation.

The applications can be extended to other SINS/multi-sensor integrated navigation or rigid body motion control simulations. When the position, velocity and attitude data at discrete time epoches are given, a continuous smooth six-degrees-of-freedom (6DOF) kinematic or dynamic trajectory can always be generated through the dual quaternion interpolation.

## Figures and Tables

**Table 1:** The computation time of the three algorithms.

Algorithm Proposed in This Paper	Algorithm Based on Inverse SINS	Algorithm Based on Quaternion
784.74 s	813.14 s	750.92 s

## Abbreviations

The following abbreviations are used in this manuscript:

UAV	Unmanned aerial vehicles
GNSS	Global navigation satellite system
IMU	Inertial measurement unit
SINS	Strapdown inertial navigation system
DQ	Dual quaternions
ECI	Earth-centered inertial
DOF	Degrees of freedom

## Appendix A. Derivation of (2)

The rotation can be described by angular rates $\omega_{ib}^{b}$ , and $\omega_{ib}^{b}$ can be calculated by $\omega_{ib}^{b}=2q^{*}\circ \dot{q}$ , where q is the real part of the dual quaternion $\overset{⌢}{\;q}$.

The translation can be described by specific force $f_{ib}^{b}$. Using the relationship between $\omega_{ib}^{b}$ and q as reference, the following is obtained

(A1) $$\begin{array}{cc} {\overset{⌢}{\;\omega }}_{ib}^{b} & =2{\overset{⌢}{q}}^{*}\circ \dot{\overset{⌢}{\;q}}=2{\left(q+\varepsilon p\right)}^{*}\circ (\dot{q}+\varepsilon \dot{p})=2(q^{*}+\varepsilon p^{*})\circ (\dot{q}+\varepsilon \dot{p})\\ & =2q^{*}\circ \dot{q}+2\varepsilon (q^{*}\circ \dot{p}+p^{*}\circ \dot{q})\\ & =\omega_{ib}^{b}+\varepsilon f_{ib}^{b}\\ & =\omega_{ib}^{b}+\varepsilon ({\dot{t}}^{b}+\omega_{ib}^{b}\times t^{b}) \end{array}$$

(A2) $$\begin{array}{cc} f_{ib}^{b} & =2\left(q^{*}\circ \dot{p}+p^{*}\circ \dot{q}\right)={\dot{t}}^{b}+\omega_{ib}^{b}\times t^{b}\\ & =q^{*}\circ f_{ib}^{i}\circ q=q^{*}\circ \left(a_{ib}^{i}-g_{\mathrm{ib}}^{i}\right)\circ q \end{array}$$

(A3) $$\begin{array}{cc} {\dot{t}}^{b} & =f_{ib}^{b}-\omega_{ib}^{b}\times t^{b}=\left(a_{ib}^{b}-g_{ib}^{b}\right)-\left[\omega_{ib}^{b}\times \right]C_{i}^{b}t^{i}\\ & =C_{i}^{b}\left(a_{ib}^{i}-g_{ib}^{i}\right)-\left[\omega_{ib}^{b}\times \right]C_{i}^{b}t^{i} \end{array}$$

Substituting ${\dot{C}}_{b}^{i}=C_{b}^{i}\left[\omega_{ib}^{b}\times \right]$ and ${\dot{C}}_{i}^{b}=-\left[\omega_{ib}^{b}\times \right]C_{i}^{b}$ in the above formula, the following is obtained:

(A4) $${\dot{t}}^{b}=C_{i}^{b}\left(a_{ib}^{i}-g_{ib}^{i}\right)+{\dot{C}}_{i}^{b}t^{i}$$

If A=BC , according to the rule we have $\dot{A}=\dot{B}C+B\dot{C}$. For the above formula, we can rewrite it as

(A5) $${\dot{t}}^{b}={\dot{C}}_{i}^{b}t^{i}+C_{i}^{b}{\dot{t}}^{i}$$

(A6) $${\dot{t}}^{i}=a_{ib}^{i}-g_{ib}^{i}$$

(A7) $$t^{i}=\int_{0}^{t}\left(a_{ib}^{i}-g_{ib}^{i}\right)dt=v_{ib}^{i}-\int_{0}^{t}g_{ib}^{i}dt$$
